# Development and validation of speech‐based biomarkers for measuring clinical progression in AD clinical trials

**DOI:** 10.1002/alz70856_103297

**Published:** 2025-12-26

**Authors:** Michael J. Spilka, Mengdan Xu, Balazs Toth, Somaye Hashemifar, Rainier Amora, Jessica Robin, Edmond Teng, Cecilia Monteiro, William Simpson

**Affiliations:** ^1^ Winterlight Labs (Cambridge Cognition), Toronto, ON, Canada; ^2^ Genentech, Inc., South San Francisco, CA, USA

## Abstract

**Background:**

Progressive language changes are evident in Alzheimer's disease (AD). Natural Language Processing (NLP) tools can provide more objective measurement of language impairment and facilitate the use of speech biomarkers for tracking clinical progression. We evaluated and compared several digital speech‐based markers developed from recordings from two phase 2 clinical trials.

**Method:**

Recordings of the Clinical Dementia Rating (CDR) interview were analyzed for 227 English‐speaking participants enrolled in two phase 2 trials of semorinemab: Tauriel (MCI‐to‐mild AD; NCT03289143) and Lauriet (mild‐to‐moderate AD; NCT03828747). Only placebo arm data was used for the Lauriet trial due to a treatment effect on the ADAS‐Cog11. Acoustic and linguistic speech features were calculated from patients’ responses to the “recent experience” CDR prompt. Data were split ∼60/40% into training and test sets for development and validation of speech composite scores. We evaluated three feature selection approaches: 1) features from our previously published speech biomarker (Robin et al., 2023; 9 features), 2) features showing a stringent significant (*p* <0.001) effect of change over time (12 features), and 3) features with a significant (*p* <0.05) effect of time in addition to greater clinical interpretability (e.g., linguistic vs. signal processing features; 18 features). Speech composites were evaluated on longitudinal change, test‐retest reliability, and correlations with clinical endpoints (ADAS‐Cog11, CDR‐SB, ADCS‐ADL, MMSE).

**Result:**

Within the test set, all three composites showed significant change over time, with baseline to endpoint effect sizes (Cohen's *d*) ranging from 0.47‐0.59. Screening vs. baseline test‐retest reliability was adequate (intraclass correlations ranging from 0.67‐0.80). All three composites were significantly and similarly correlated with clinical endpoints at baseline (Spearman rho ranging from |0.21‐0.49|).

**Conclusion:**

Speech characteristics can be combined into meaningful indices of disease progression across the spectrum of MCI to moderate AD. All three composites performed well overall. The best performing composite was our previously published Tauriel derived biomarker, which had the largest effect size of change, highest test‐retest reliability, and was also the most parsimonious measure with the fewest features. These results highlight the potential utility of a speech‐based biomarker as an objective and low‐burden measure of clinical progression to complement traditional endpoints in AD clinical trials.

